# Efficacy of non-steroidal anti-inflammatory drug monotherapy in children with systemic juvenile idiopathic arthritis

**DOI:** 10.1186/1546-0096-10-S1-A54

**Published:** 2012-07-13

**Authors:** Meredith P Riebschleger, Jasmine Stannard, Matthew M Davis, Sarah J Clark, Barbara S Adams

**Affiliations:** 1 University of Michigan, Ann Arbor, MI, USA

## Purpose

Treatment of systemic juvenile idiopathic arthritis (sJIA) is one of the most challenging tasks faced by pediatric rheumatologists. Traditionally, many pediatric rheumatologists have used a “pyramid” approach, starting with non-steroidal anti-inflammatory drug (NSAID) monotherapy prior to the initiation of disease modifying anti-rheumatic drugs (DMARDs) or biologic agents. Despite the widespread use of NSAID monotherapy, the likelihood of response to this regimen has not been well characterized. In addition, some clinicians have recently begun to advocate for the use of biologic agents as first-line therapy for all patients with sJIA, despite the risks of patient discomfort, infection, and malignancy that may be associated with their use. The goals of this study are (1) to determine the frequency of clinical response to NSAID monotherapy in sJIA, and (2) to identify factors that predict that response.

## Methods

The authors conducted a single-center cohort study of children aged 0-18 years who were diagnosed with sJIA between January 1, 2000, and December 31, 2009. Children who met International League of Associations for Rheumatology (ILAR) criteria for sJIA and had at least 4 months of follow-up from the time of initial diagnosis were included in the study. An abbreviated preliminary review of electronic medical records was used to determine the frequency of NSAID monotherapy trial and overall clinical outcomes (remission versus lack of response). Complete chart review is ongoing and includes identification of: (1) patient demographics; (2) characteristics of initial presentation, including medical history, physical exam, and laboratory findings; (3) detailed therapeutic regimens; and (4) clinical outcomes.

## Results

56 children were eligible for inclusion in the cohort. Forty children (71%) underwent an initial trial of NSAID monotherapy. The other 16 children received additional drugs, most often including DMARDs and/or steroids within one week of diagnosis. Twelve of the children who received NSAID monotherapy (30%) experienced clinical remission (symptom-free on medication) without escalation of their medication regimen and seven of those (18%) were able to discontinue their NSAID, thereby achieving complete remission (symptom-free off medication). After full chart review has been completed for all patients in the cohort, logistic regression will be used to determine whether characteristics present at the time of initial diagnosis can predict the likelihood of remission with NSAID monotherapy.

## Conclusion

NSAID monotherapy appears to be effective in achieving complete remission in a subset of children with sJIA. Our findings may help identify which children should receive a trial of NSAID monotherapy, as opposed to proceeding directly to more aggressive treatment including DMARDs and biologic agents.

## Disclosure

Meredith P. Riebschleger: None; Jasmine Stannard: None; Matthew M. Davis: None; Sarah J. Clark: None; Barbara S. Adams: None.

